# “We need a one-stop-shop”: co-creating the model of care for a multidisciplinary memory clinic with community members, GPs, aged care workers, service providers, and policy-makers

**DOI:** 10.1186/s12877-019-1410-x

**Published:** 2020-02-11

**Authors:** Genevieve Z. Steiner, Carolyn Ee, Shamieka Dubois, Freya MacMillan, Emma S. George, Kate A. McBride, Diana Karamacoska, Keith McDonald, Anne Harley, Gamze Abramov, Elana R. Andrews-Marney, Adele E. Cave, Mark I. Hohenberg

**Affiliations:** 1 NICM Health Research Institute, Western Sydney University, Penrith, NSW 2751 Australia; 2 Translational Health Research Institute (THRI), Western Sydney University, Penrith, NSW 2751 Australia; 3 School of Health Sciences, Western Sydney University, Penrith, NSW 2751 Australia; 4 School of Medicine, Western Sydney University, Penrith, NSW 2751 Australia; 5 School of Psychology, University of Wollongong, Wollongong, NSW 2522 Australia; 6 South Western Sydney Primary Health Network (SWSPHN, Campbelltown, NSW 2560 Australia; 7 Campbelltown Hospital, South Western Sydney Local Health District, Campbelltown, NSW 2560 Australia

**Keywords:** Memory clinic, Model of care, Dementia, Neurocognitive disorder, Mild cognitive impairment (MCI), Qualitative, Co-design

## Abstract

**Background:**

Timely diagnosis of dementia has a wide range of benefits including reduced hospital emergency department presentations, admissions and inpatient length of stay, and improved quality of life for patients and their carers by facilitating access to treatments that reduce symptoms, and allow time to plan for the future. Memory clinics can provide such services, however there is no 'gold standard' model of care. This study involved the co-creation of a model of care for a new multidisciplinary memory clinic with local community members, General Practitioners (GPs), policy-makers, community aged care workers, and service providers.

**Methods:**

Data collection comprised semi-structured interviews (*N* = 98) with 20 GPs, and three 2-h community forums involving 53 seniors and community/local government representatives, and 25 community healthcare workers. Interviews and community forums were audio-recorded, transcribed verbatim, and coded by thematic analysis using Quirkos.

**Results:**

GPs’ attitudes towards their role in assessing people with dementia varied. Many GPs reported that they found it useful for patients to have a diagnosis of dementia, but required support from secondary care to make the diagnosis and assist with subsequent management. Community forum participants felt they had a good knowledge of available dementia resources and services, but noted that these were highly fragmented and needed to be easier to navigate for the patient/carer via a 'one-stop-shop' and the provision of a dementia key worker. Expectations for the services and features of a new memory clinic included diagnostic services, rapid referrals, case management, education, legal services, culturally sensitive and appropriate services, allied health, research participation opportunities, and clear communication with GPs. Participants described several barriers to memory clinic utilisation including transportation access, funding, awareness, and costs.

**Conclusion:**

This study demonstrates the importance of working with stakeholders to co-design models of care for people with dementia that take into account the local communities’ needs. Findings pave the way for the development of a potential new “gold standard” memory clinic model of care and operationalise new national clinical guidelines.

## Background

Dementia is a debilitating syndrome characterised by a decline in cognition that interferes with function and independence, with an aetiology spanning more than 100 diseases [1]. Worldwide, dementia is estimated to affect 50 million people, and by 2050 this number is expected to increase to over 131 million people [2]. Dementia currently costs $818 billion USD worldwide and will soon outstrip spending on any other health condition [3].

Timely diagnosis (when patients initially present to clinicians with complaints of cognitive decline, regardless of disease stage and severity [4]) results in better outcomes for people with dementia, their families, and the health care sector [5, 6]. Although there is no cure for dementia, there is an emerging body of evidence which demonstrates that early diagnosis is beneficial [7] and cost-effective for the health care system [8]. Timely access to a specialised cognitive assessment service and early diagnosis facilitates: access to treatments which reduce cognitive and neuropsychiatric symptoms [9]; reduces emergency department presentations from residential aged care facilities [10], hospital admissions [11] and length of stay [12, 13]; improves medication adherence and monitoring [11], advance care planning [14], and access to community-based services to support activities of daily living (ADLs); and allows people to stay at home for longer [4, 15]; results in improved mental health for carers [16] and greater acceptance of the diagnosis for both patients and families [17]. This is particularly relevant during the symptomatic pre-dementia mild cognitive impairment (MCI) phase which confers significantly increased dementia risk [18, 19], as its classification as a diagnostic entity has been fraught with difficulty due to the lack of a consensus upon clear clinical and research diagnostic and management guidelines; significant efforts have been made to clarify this in recent times [20–22].

In Australia (the setting for the present study), the 2016 National Health and Medical Research Council (NHMRC) Clinical Practice Guidelines and Principles of Care for People with Dementia [23] made a series of key recommendations emphasising integrated care (which is coordinated between primary, secondary, and tertiary levels of care), including referral to memory assessment specialists, person-centred care with access to a care coordinator, specialised dementia care training for clinical staff, and community-based services to promote independence in activities of daily living. Such recommendations are made in similar international guidelines [24]. Memory clinics can facilitate the operationalisation of these guidelines as they are specialised dementia centres that can provide a service for people with dementia and their families to facilitate timely diagnosis, the associated benefits, and support dementia management.

Any innovation in dementia care must address the needs of General Practitioners (GPs). GPs see over 85% of the Australian population each year, 27.8% of which are people over 65 years [25]. GPs are ideally placed to identify dementia and assist with the implementation of the NHMRC’s recommendations [23], however, it has been found that GPs may fail to identify around 50% of early dementia cases [26]. GPs have identified inadequate training in dementia management and time pressures as barriers for their identification and management of dementia [27, 28]. There is an urgent need for GP input into improving the diagnosis and management of dementia, and for increased GP support.

The importance of consumer and community involvement in health and medical research has recently been emphasised by the NHMRC by the release of a revised statement [29]. The 2016 statement acknowledges the essential role of the community as contributors, participants, and advocates in health care, and emphasises the need for community engagement in health and medical research. Researchers and research organisations are strongly encouraged to engage consumers and community members in research design, conduct, and translation, with the vision of improving the health and wellbeing of Australians via research.

### Study setting and aims

As the largest and fastest growing district in Metropolitan Sydney, South Western Sydney houses more than 850,000 people across seven local government areas [30], with the population predicted to exceed one million by 2021 [31]. This region is also culturally and lingually diverse, with 38% of residents born overseas and 42% of households in Greater Western Sydney speaking a language other than English [32]. It is anticipated that South Western Sydney will see the biggest increase in dementia prevalence rates in the state of New South Wales, Australia (up to 460% by 2050) [33], with the vast majority of these residents not having access to a memory clinic.

In line with the NHMRC’s recommendations [23, 29], the growing evidence base supporting the benefits of an early diagnosis [7, 9, 14–17], and preliminary research identifying gaps in current dementia services in South Western Sydney [34], this study sought to design a new multidisciplinary memory clinic service. The aim of this study was to ensure the region-specific needs of the memory clinic were considered by co-creating the model of care with local GPs, community health care workers, local government, and local community members including seniors, carers, and people with dementia.

## Methods

### Recruitment and participants

A purposive sampling strategy was utilised to recruit GPs from South Western Sydney, community health care workers, and community members. Study flyers were distributed by email via South Western Sydney Primary Health Network (SWSPHN; Primary Health Networks are an Australian Federal Government initiative that provide support to primary care practitioners, facilitate patient access to primary care, and support integrated care), consumer networks and social media, and the study was promoted online and/or in local newspapers by three local councils (Camden Council, Campbelltown City Council, and Liverpool City Council). The study was also promoted by the research team to GPs at Continuing Professional Development (CPD) events facilitated by SWSPHN. Recruitment of GPs took place over an 8-month time frame (June 2017–February 2018), and community forums over 3-months (July–October 2017). Upon receiving promotional material, potential participants contacted the study’s research assistant to register their interest in participating in the study. The final sample (*N* = 98) included 20 GPs, 53 seniors and community/local government area representatives, and 25 community health workers; there were no withdrawals. This sample size was sufficient to reach data saturation [35]. Ethical approval was obtained from Western Sydney University Human Research Ethics Committee (H12091).

### Data collection and analysis

All participants were provided with a copy of the participant information sheet and consent form, and informed consent was obtained prior to commencing data collection: verbal consent was obtained for GPs as these interviews were conducted via telephone, and written consent was obtained for community forums as these were conducted face-to-face. Community forums were chaired by an experienced female facilitator and senior research fellow (GZS) and female assistant (DK), and GP interviews were conducted by an experienced male research assistant with an educational background in psychology (BPsych. graduate). No repeat interviews were conducted, and transcripts were not checked by participants.

### GPs

In-depth semi-structured telephone interviews of ~ 30 min in length were conducted between June 2017 and February 2018. Demographic information was collected from GPs and an interview schedule (see Additional file 1) was used that was grouped into the following three themes: assessment of patients with cognitive impairment, current dementia services and resources, and expectations for a new memory clinic service.

### Community health care workers and community representatives

Three community forums hosted by Camden Council, Campbelltown City Council, and Liverpool City Council were run as large focus groups of ~ 2 h in length. Community health care workers, community representatives (seniors, carers, and people with dementia), and representatives from local government participated in the three forums during October–November 2017. An interview schedule (similar to that used for GPs; see Additional file 1) was utilised and grouped into the following two themes: needs and barriers of current dementia services and resources, and expectations for a new memory clinic service.

### Data analysis

All GP interviews and community forums were audio recorded and transcribed verbatim. Any potentially identifiable information was removed, and pseudonyms used throughout. Transcripts from both data sources underwent thematic (inductive) analysis [36]. Transcripts were coded in Quirkos v.1.4.1 software using the method of constant comparison, which takes an inductive approach. This iterative process consisted of systematically identifying, comparing and coding themes within and between the interviews. Emerging categories and associations among the codes led to the development of several themes and sub-themes. Analytical rigour was achieved by independent review of all transcripts and coded data by two members of the research team (SD, GZS). Discrepancies in coding were discussed between the research team until consensus was met.

## Results

### GP demographics

GPs (*N* = 20) were 11 males, 9 females, mean age = 47.4 years (*SD* = 12.2), who had been practising as a GP for a mean of 17.0 years (*SD* = 12.5), and were from 18 different practices across South Western Sydney. GPs saw an average of 4.8 (*SD* = 7.1) patients/week with dementia, 6.2 (*SD* = 7.2) patients/week with MCI, and referred 2.0 (*SD* = 4.5) patients/week to secondary care for MCI or dementia.

### Community forum demographics

Community forum participants were 14 males and 64 females. Participants included 46 members of the community (seniors, residents, people with dementia, and carers), 15 community service providers (including residential aged care and Dementia Advisory Service), 10 nursing, allied health, and staff specialists, 5 local government representatives, and 2 community group representatives (i.e., local community groups representing seniors’ interests).

### Qualitative data

Across all data sources, findings are reported within three overarching themes: (1) GPs’ attitudes towards the diagnosis and management of MCI and dementia, (2) perceptions and attitudes towards local dementia resources and services, and (3) expectations for a new memory clinic.

#### Theme 1: GPs’ attitudes towards the diagnosis and management of cognitive impairment and dementia

Table 1 details the three subthemes and supporting excerpts for Theme 1. Content from Theme 1 emerged from GP interviews only.

**Table 1:** Theme 1: GPs’ attitudes towards the diagnosis and management of cognitive impairment and dementia

Sub-themes	Example excerpts highlighting sub-theme meaning	Excerpt number relating to text
Assessment of older patients for cognitive impairment		
	“Why do I do it? Just part of your annual check-up, part of your sort of annual assessment. Everybody over 75 gets it once a year, and you do like cognitive assessment, whether or not they might have had any falls, you know, what’s happening at home, social assessment, sort of a general health assessment, basically an assessment of their nutrition.” – 2GP	1.1a
	“Not as a screen, but I do see a lot, you know if there’s a trigger factor for it, like patients might have forgotten their appointment or they don’t seem to be taking their medications properly, or their spouse comes in with them and mentions trouble. Yeah, so I don’t have a system for running a cognitive screen on people.” – 12GP	1.1b
	‘Patients come in and there are multiple complex [problems] these days and people want me to do ten problems in 15 min, so there’s not time.’ – 3GP	1.1c
Perceptions on the utility of a diagnosis of MCI or dementia		
	‘Well I think the most important thing is to explain to the patient what’s going on with them so the patient is aware of the fact that you believe there’s a process taking place in their blame that is the reason why they’re having the symptoms they’re having, and explaining that to their family as well, so then you can treat them most effectively to manage the various aspects of the dementia.’ – 10GP	1.2a
	‘It gives a name to what’s happening to them if they’ve noticed symptoms and a justification for any deficits they’re noticing. It also gives them time. If it’s mild cognitive impairment they’ve got time to put in strategies and to make some plans for the future in an informed way, which they can’t if it isn’t addressed.’ – 11GP	1.2b
	‘And it certainly can be somewhat distressing for some people to feel that their mind’s not working as well as it should and certainly with mild cognitive impairment it does seem as though some people may show signs of that but not necessarily go on to dementia, and also that some processes are much slower or faster than others, so I find it a little bit of a vexed question when it seems at the early stages.’ – 6GP	1.2d
	‘It is difficult because we don’t have that many treatments that are very effective, and so that it’s hard to diagnose something and then say “sorry, we haven’t got much we can do for you.”’ – 6GP	1.2e
Assistance with diagnosis and management of dementia		
	‘I personally wouldn’t want to diagnose myself, I would want them to have like more testing and at least a CT scan in secondary care to try and make the diagnosis.’ – 9GP	1.3a
	‘Yes, yeah. I think I feel that, I think I can make the diagnosis myself but – because it’s usually fairly clear when you do the assessment, but just for more, again for more detail on the cognitive scale … and also takes a little bit more time and specialists will have that.’ – 3GP	1.3c
	‘We do require the services of specialists under the Commonwealth system requiring specialists to institute treatment which is pharmacological, pharmacotherapy for dementia, so clearly we need to refer for accessing treatments for dementia, pharmacological treatments that is.’ – 10GP	1.3d
	‘Well dementia definitely, yes. If they’ve got dementia I do get that diagnosis confirmed by a specialist because I think that’s very important. With mild cognitive impairment well yes, I do yeah. Well yes, I make that diagnosis, yes.’ – 21GP	1.3e
	‘Yeah, I think the management is the problem with – because obviously these people need a lot more support. I think the places they could go to that deal specifically with that, it would be much better, yeah.’ – 2GP	1.3f

##### Assessment of older patients for cognitive impairment

GPs’ assessment of cognitive impairment in older patients varied. GPs who regularly assessed for cognitive impairment did so as part of patients’ annual health assessments, or felt that it was necessary because of the prevalence of cognitive impairment in older patients (excerpt 1.1a). Some other GPs felt that the decision to assess for cognitive impairment was dependent on the signs and symptoms of the patient (excerpt 1.1b). GPs who did not regularly assess for cognitive impairment gave reasons including lack of time (excerpt 1.1c) or the patient’s unwillingness due to fear of the results (excerpt 1.1d).

*“Yes, there’s no time and I think lack of public interest in it, and also probably personal fear about a diagnosis like that to be honest. I mean they’re probably the two main barriers, three main barriers.”* – 3GP (excerpt 1.1d)

##### Perceptions on the utility of a diagnosis of MCI or dementia

Nearly all GPs felt it was useful for patients to have a diagnosis of MCI or dementia. Reasons for this belief included: informing families of the condition (excerpt 1.2a), beginning advance care planning (excerpt 1.2b), and commencing management and treatment that may provide the opportunity to slow down the progression of the disease (excerpt 1.2c). The GPs who did not find it useful stated that this was because a diagnosis can be distressing for the patient (excerpt 1.2d), or because they felt there was no point given that there is no cure for dementia (excerpt 1.2e).

*“I would definitely say so because early diagnosis means that they have more time to sort out their affairs, they have more time to manage the condition if it could be managed or slowed down, so all in all I think early diagnosis would definitely be beneficial.”* – 9GP (excerpt 1.2c)

##### Assistance with diagnosis and management of dementia

Most of the GPs stated that they required assistance with the process of *diagnosis* of MCI or dementia, with many referring patients to secondary care to assist with this (excerpt 1.3a). Reasons for GPs not diagnosing MCI or dementia included a preference for confirmation from a specialist to avoid the potential ramifications associated with making the diagnosis (excerpt 1.3b), lack of time (excerpt 1.3c), and the perception that only specialists are able to prescribe dementia medications (excerpt 1.3d). Some GPs felt capable of diagnosing MCI or dementia themselves; this was more so for the milder cases (excerpt 1.3e). Furthermore, some GPs also felt that they required assistance with the *management* of patients with MCI or dementia (excerpt 1.3f).

*“I don’t personally make the final diagnosis. I’m much more comfortable kind of getting – like you know, if there’s a strong suspicion it’s dementia and usually I mean usually I’m right, I usually would just go to the geriatrician to make the final diagnosis just because it’s such a, it’s such a big diagnosis to make, it’s got a lot of ramifications, and I’d rather have like a specialist kind of give the final say about that, yeah.”* – 4GP (excerpt 1.3b)

#### Theme 2: perceptions and attitudes towards local dementia resources and services

The three subthemes and excerpts for Theme 2 are detailed in Table 2 and emerged from all data sources; variation in content from different data sources is specified below.

**Table 2:** Theme 2: Perceptions and attitudes towards local dementia resources and services; CF = community forum participants

Sub-themes	Example excerpts highlighting sub-theme meaning	Excerpt number relating to text
Understanding of available resources and services		
Resources	‘I think like diagnosis there are things like screening tools, MMSEs and GPcogs and those kind of things. In terms of like general management resources, I mean there are of course like the referral pathways that we have like geriatricians, for driving assessments there’s actually the RMS can do it, which is good. There’s the home – like there’s like the ACAT assessment and then the home care, like the HACC packages, like each, whatever each thing.’ – 4GP	2.1a
	‘Yeah, so I think the thing with resources, I think it’s sort of more general and more generic resources. I think there are some good stuff that’s available already and I don’t really think we should sort of reinvent the wheel both internationally and in Australia. There’s some good sort of general resources.’ – 22GP	2.1b
Services	‘There’s lots of programs, lots of each programs and different programs which really cover a lot of things. There’s a centre of excellence at Hammondville. There’s Carrington and the other providers in the Whiddon Group. There’s Broughton House has got a really good dementia day care as well as Myrtle Cottage. The aged care assessments, there’s Alzheimer’s Australia, there’s speech therapists, podiatrists, physios, doctors and specialists. The people on the transport are really wonderful, on the train and the bus and the taxi. They’re excellent with wheelchairs and so kind. And there’s Meals on Wheels.’ – 14CF	2.1d
	‘Look, I’m actually really happy with what we’ve got, which is a secondary service that we can refer to’ – 19GP	2.1e
	‘Q. Are you familiar with any dementia specialised services in the local area? A. Not in the local area here, no.’ – 9GP	2.1f
	‘In my case I don’t have the – I don’t have any qualms in referring someone if I think that they need to be referred [to a dementia-specialised service].’ – 10GP	2.1 g
	‘Yes, there are a number, and often it’s the person themselves that doesn’t want referral. A lot of people are very independent or they don’t really want to know. If there’s anything happening they just want to soldier on, and so that often does stop us referring, or if we do refer they are not very keen to have any services involved.’ – 6GP	2.1 h
Barriers to current resources and services		
Resources	‘We would like accessible information in a format that all of us can use.’ – 14CF	2.2a
	‘I think some resources to help people navigate their way into receiving services or through that, sort of follow that pathway and receiving services and getting some direction with that I think is useful. So some more resources along those lines to help people understand the website, how to use it and you know, how to access services.’ – 10GP	2.2b
	‘I think if once a diagnosis is made, I think it would be useful for them to be given a list of various common things that they might face and who they might be able to contact to get more help with regards to that.’ – 9GP	2.2d
	‘We don’t have enough resources now.’ – 15CF	2.2e
Services	‘Cost effective specialists, because specialists cost a lot of money and there’s a long waiting list within the actual health system.’ – 14CF	2.2f
	‘A great need for all of those things, trying to navigate – yes, everything. So as you said, education, allied health, you mentioned psychology but yeah, allied health, legal. All of those things are really, really needed, and at the moment you can access them a bit but they’re fragmented all around the place and not easy for people who are trying to – you know, may not be able to drive either, not being able to get to. So in a single site would be wonderful.’ – 11GP	2.2 g
	‘We find that with all of the hospital or area health service-based services, they may be there but we’re often not informed as GPs on how to actually access them, and then they often change the access process or the requirements. Nobody actually tells us.’ – 11GP	2.2 h
	‘I think the issue with dementia-specific services is that when My Aged Care came in they decided not to make services dementia-specific, so whereas you had dementia monitoring services and those sorts of things, they’ve become just social support services.’ – 16CF	2.2i
	‘I might include some of the past comments, but we’d like to see more skilled workers who have specific training in dementia.’ – 14CF	2.2j
What resources and services should focus on		
Resources	‘I think education is very helpful for – to educate the carers as well as for patients about what’s going on.’ – 10GP	2.3a
	‘There needs – I mean we’re going back to the putting things in newspapers, but we’ve got to do whatever we can to remove the stigma of the word ‘dementia.’ If it’s out there in the community and generally talked about then I don’t think there’s quite such an issue to get somebody diagnosed. I mean if it’s as well-known out there as cancer is or any of those other sorts of things then there might be a little bit more – or little bit less resistance from people.’ – 16CF	2.3c
	‘Someone else comes in, either is not aware or doesn’t realise polypharmacy is bad and get yet another drug and another drug, and I’ve seen as many as five different antipsychotics, mood stabilisers and antidepressants for really the same behaviours.’ – 15CF	2.3d
Services	‘There’s not enough respite full stop in the area. And carers and also working carers support, there’s no support there for us because we work while support groups are on.’ – 14CF	2.3e
	‘I suppose sort of at the more pointy end, so for people who are having fairly severe dementia, particularly with sort of behavioural challenges as well for the family, practical advice and support for family in managing those behaviours.’ – 22GP	2.3f

##### Understanding of available resources and services

Most community forum participants demonstrated that they had a good knowledge of the available *resources* (e.g., information, education, screening tools) for MCI and dementia (excerpt 2.1a). Several participants (GPs and community forum participants) felt that these resources were satisfactory (excerpt 2.1b), however, GPs felt confused with how to *access* the resources that are available for dementia or were not sure of what is available (excerpt 2.1c).

Many participants (GPs and community forum participants) also demonstrated in-depth knowledge of the local dementia-specialised *services* (e.g., day care centres, memory clinics) (excerpt 2.1d), with some indicating that they were happy with these services (excerpt 2.1e). Several participants (both data sources) were unfamiliar with or had a limited understanding of local services (excerpt 2.1f). Some GPs indicated their willingness to refer to dementia-specialised services and frequently do so (excerpt 2.1 g), where others did not refer due to patients’ unwillingness for a referral, usually due to denial of their condition (excerpt 2.1 h).

*“There just seems to be so much information out there and we deal with so many different types of illnesses in general practices, and we have guidelines and pathways for every different one. It’s often hard to keep them in your head certainly, and so I don’t know whether something could be put on maybe Health Pathways*^1^ *or something that we can access easily and know where to look.”* – 6GP (excerpt 2.1c)

##### Barriers to current resources and services

Many GPs and community forum participants articulated that current *resources* for MCI and dementia need to be improved. A need was expressed for resources that are clear and easily accessible (excerpt 2.2a), and easy to navigate for the person with dementia and/or carer (excerpt 2.2b). Ideas to improve navigation included creating a single point of contact for carers and patients to use (excerpt 2.2c), or an information summary sheet with a list of all available resources (excerpt 2.2d). Many of the participants felt that there was a need for more resources in general for patients, families, and GPs (excerpt 2.2e).

Both GPs and community forum participants described problems with current local dementia *services*. Barriers to service access discussed included long waiting times and cost (excerpt 2.2f), and fragmentation resulting in services being difficult to access and navigate for patients, families, and GPs (excerpt 2.2 g). GPs also highlighted that there was poor communication between dementia services and themselves and that they required a clearer referral pathway as they were often unsure of which services to refer patients to (excerpt 2.2 h). GPs and community forum participants described an overall lack of dementia-specific services (excerpt 2.2i), and staff with specialised dementia training (excerpt 2.2j); some also highlighted a lack of after-hours services.

*“One number. That’s one, just one phone number. It’s too difficult for elderly people to navigate their way through the maze of aged care.”* – 15CF (excerpt 2.2c)

##### What resources and services should focus on

GPs and community forum participants described a number of *resources* that they felt were helpful and/or would like to see more of. These included educational resources for GPs, seniors, people with dementia, and carers regarding what the early warning signs are, what to expect after a diagnosis, how to approach caring for somebody with dementia, and lists of available services (excerpts 2.3a and 2.3b). Community forum participants also felt that improving the general public’s dementia literacy through dementia awareness campaigns would help to breakdown the stigma associated with a diagnosis and increase the likeliness for people to seek help (excerpt 2.3c). Further, GPs and community forum participants also articulated a need for polypharmacy education for GPs and aged-care workers to prevent the misuse of medications (excerpt 2.3d).

*“In the beginning stages, we feel that it’s family members who have to refer to the GPs before they get any sort of diagnosis at all, and it would help if we knew a bit earlier what to look for. People who are on their own feel that they have to work really hard to get any support because they don’t really know where to go. It’s often hidden from GPs unless relatives or very close friends speak of what the problems are. Some of these people in the beginning stages can hide it very well, and we do need an assessment criteria in our homes when we’re talking to our friends, so oh yes, perhaps I’d better have a look at that or be referred for that.”* – 14CF (excerpt 2.3b)

The majority of participants (from both data sources) described a need for more dementia-specialised *services*. These were an increase in services for carer support and respite, services that support people with MCI or dementia (including home care services for those who wished to remain at home; e.g., community transport, cleaning services, medication assistance), and services to help families cope with challenging behaviours (excerpts 2.3e and 2.3f). Case management was also highlighted as an important service area by GPs and community forum participants (excerpt 2.3 g).

*“We’d like to bring back case management with guided referrals, and case managers to do complex care plans so that it’s not the onus of the carer to try and navigate their way around services that should be. A case manager that takes that concern off the carer and helps them with guided referrals.”* – 14CF (excerpt 2.3 g)

### Theme 3: expectations for a new memory clinic

Table 3 shows the five subthemes and excerpts that were identified for theme 3 from all data sources; as above, content emerging from different data sources is delineated.

**Table 3:** Theme 3: Expectations for a new memory clinic; CF = community forum participants

Sub-themes	Example excerpts highlighting sub-theme meaning	Excerpt number relating to text
A new memory clinic could optimise patient care		
	‘I’m not sure what there is in terms of provision of services. I know that there’s – there are some day centres around and suchlike things, and there are some things available via packages, but just having – either having the resource from the local area health to tell us what is available and where it is would be really helpful because we just don’t – haven’t had any communication for a very long time about what there is and how to get to it.’ – 11GP	3.1a
	‘They can go in there and we never hear anything again and there’s no – there’s a lack of communication. The community is very one-sided you know, like you’re involved initially as a patient and then we hear nothing back about what has been done or whatever … I think that anything that’s going to be successful I think will require you know engaging with GPs and communication with GPs. I think that’s really important so that, you know.’ – 2GP	3.1b
	‘I think if it has precise and as much information that they can give about the process and everything would be useful, and just to keep us informed about how things are progressing and when to expect these things to start, so information transfer would be useful at this stage, yes.’ – 9GP	3.1c
Expected services provided		
Specialised diagnostic services	‘To a memory clinic, I support what I would use a memory clinic for … is for there to be a quite clear and definitive diagnosis. So if it is dementia, clearly dementia, and hopefully some information about – and prognosis as well.’ – 10GP	3.2a
Rapid access to care and case management	‘I suppose if you want to prioritise them, rapid access would probably be top of the list, and then certainly case management’ – 23GP	3.2b
Allied health	‘The allied health as well, I think that would be helpful in terms of like getting the most out of it.’ – 13GP	3.2c
Support groups	‘I’m thinking that rather than counselling, probably more support type groups might be more useful for carers and patients and gender assessing for groups for patients. That sort of support group type counselling rather than individual counselling I think.’ – 2GP	3.2d
Expected features of the clinic		
Easily accessible	‘We also feel that the main building for the memory clinic needs to be extremely accessible, it needs to have parking, it needs to be accessible by public transport.’ – 14CF	3.3c
Culturally sensitive	‘I think that if there were a local service, I’d feel more confident to send them to a local service that I know has also a multicultural sort of staffing or approach because yeah, the people that are most like hmmm are people who – English is their second language, they have a tendency to minimise their symptoms and sort of get on with things, so I would be less likely to send them to a clinic that I felt was just going to see them like a regular patient as opposed to a clinic that was like yeah, also we have people who can like you know connect on that like cultural and language level.’ – 13GP	3.3d
Barriers to the clinic		
Accessibility	‘Because that’s why I’m thinking that you know, if there was like a pick- up community bus thing that picked all these people up to go to these services, it would be easier I think.’ – 2GP	3.4b
Awareness of the clinic	‘So they wouldn’t be able to – I suppose another barrier is that GPs have no idea about this clinic. They have no idea, they don’t know anything about it so they’re not going to refer.’ – 15CF	3.4c
Referrals and costs		
Referral process	‘You know, something that’s fairly quick so that I know it’s being organised and the patients will be seen’ – 1GP	3.5b
	‘A pro forma, yeah, so that you kind of – sort of you kind of can narrow down what information you need from us, right, so it’s easier, and then we know what you want so we can write it down.’ – 5GP	3.5c
	‘In this day and age a lot of people probably like to email or fax, or occasionally some people like faxing, sending the referral to the specialist.’ – 10GP	3.5d
Referral criteria	‘By the time you get to severe they’re often no longer you know, in general practice area because they’re more in a nursing home. So mild to moderate for diagnosis and management, particularly when they’re out in the community. There’s such a great need.’ – 11GP	3.5f
	‘I think anybody with memory problems. It’s a memory clinic isn’t it?’ – 21GP	3.5 g
Cost	‘Most people at that stage are not financially well off, so if it was bulk billable or covered by some sort of fund it would be very helpful.’ – 11GP	3.5 h

#### A new memory clinic could optimise patient care

Many of the GPs stated that a service like a new memory clinic could be valuable in optimising patient care. For example, GPs highlighted a current lack of communication between current dementia services and their practices, resulting in a lack of awareness of available services for their patients (excerpt 3.1a). GPs also highlighted the desire for a new memory clinic service to continue communication with GPs after the referral of a patient to keep them informed of the patient’s situation (excerpt 3.1b). In addition, GPs stated that it would be valuable if they were given a list of the memory clinic’s services as they would then be more likely to refer to a new clinic (excerpt 3.1c).

#### Expected services provided

Participants were asked about what services they would expect from a memory clinic (prompts were provided regarding potential services), and which ones they felt had the most value. Nearly all GPs and community forum participants expected that a new memory clinic should provide specialised diagnostic services, including early and definitive diagnoses (excerpt 3.2a). Other services with perceived value included: rapid access to care via shorter referral processes and decreased waiting lists, a case management service that ensured continuity of care (excerpt 3.2b). Participants discussed the need for a new clinic to offer educational resources and services for GPs, patients, carers and families. Additional services included allied health (particularly occupational therapists and physiotherapists), support groups for both patients and carers, links to community services, legal assistance to facilitate discussions around power of attorney and guardianship, psychological supporting including counselling to help patients and families come to terms with a diagnosis, and advance care planning (excerpts 3.2c, 3.2d, and 3.2e). Participants also highlighted the value of integrative therapies including lifestyle and mind and body activities (e.g., tai chi) to improve physical and mental health.

*“Okay, so it’s the ability to have good access to diagnostic services and treatment, early treatment for those patients that require it started, and there would also be – obviously education is extremely important always for the carers as well as for patients, and I think the ability to link into community services is part of that, but it’s sort of broader than just education, it’s more specifically giving the patients and the families a good idea of what is available online as resources as well as locally the various resources that are available.”* – 10GP (excerpt 3.2e)

#### Expected features of the clinic

Participants described the key features that they felt a new memory clinic should have. These included a multidisciplinary team, a “one-stop-shop” that offered a range of services and activities for people with MCI or dementia, and a key worker that people with dementia and families could contact (excerpt 3.3a and 3.3b). Participants also stated that the clinic should be easily accessible (public transport and sufficient parking), demonstrate cultural sensitivity via multicultural and/or bilingual workers, provide or have access to an outreach team for families at home, and offer research participation opportunities such as clinical trials (excerpts 3.3c and 3.3d).

*“And then in terms of what should be on offer, obviously having a clinician if we’re thinking about diagnosis side of things, but even having some sort of plan in terms of prevention to make it seem – I mean in a true multidisciplinary service, things like having a speech path review or having an OT or physio, having those kinds of – a one stop shop type arrangement would be highly beneficial.”* – 7GP (excerpt 3.3a)

*“I think at the very beginning people don’t want to hear any of the information that we have, but if we can connect that key person at that point and say this is your go to person and these are the things that you should be doing and outline them, I think that that will be – when they take it all in and it overwhelms them, at least they have that key person that they say this is my contact, this is my link at the clinic to support them”* – 16CF (excerpt 3.3b)

#### Barriers to the clinic

Potential issues and barriers to a new memory clinic service were discussed. Accessibility was a common barrier, particularly in relation to potential location, transport and parking options (excerpt 3.4a). Participants suggested that a free shuttle bus which operated between the clinic and patients’ homes might overcome this potential barrier (excerpt 3.4b). Additional concerns included initial and ongoing funding for the clinic’s operations, ensuring GPs and patients were aware of the memory clinic’s existence, and potentially long waiting lists (excerpt 3.4c).

*“Access to public transport, so if you’ve got people who are in those outlying areas, being able to access this service is a real issue.”* – 14CF (excerpt 3.4a)

#### Referrals and costs

Nearly all of GPs indicated that they would be happy to refer patients to a local memory clinic service if they had access to it, and that it would have value for them and their patients as a “one-stop-shop” for MCI and dementia (excerpt 3.5a). Two GPs (located in the Bowral local government area) stated that they already had access to a local memory clinic and were willing to refer patients there, and one GP was unsure if they would refer on as they felt that current dementia services were adequate.

*“It’s basically to try and get everything done under one roof and sort of be given what the thought process is, what’s the outcome of it, all in one place and given a sense of direction, and it will mean that the patient is not running around and getting a bit confused with where things are that go and with what. So I think overall in terms of patient care I think that will definitely be useful.”* – 9GP (excerpt 3.5a)

When asked about the ideal referral process, most GPs described the need for convenience and minimal time (excerpt 3.5b), and indicated their preference for a proforma or template that auto-populated patient details (excerpt 3.5c), where others stated that they would prefer a formal written specialist referral. The most frequently nominated referral submission process was via fax or email (excerpt 3.5d), with one GP requesting an online referral form. Some GPs also indicated willingness to refer via telephone.

GPs discussed their expected referral criteria for the proposed memory clinic. Many of the GPs stated that it would be most useful to refer patients with mild to moderate dementia (excerpt 3.5e), whilst others indicated that the memory clinic would not be useful for patients with severe/advanced dementia due to the higher level of care required (excerpt 3.5f). A few of the GPs said that they would like to refer all patients with MCI or dementia (excerpt 3.5 g). Many of the participants (all data sources) felt that it was important that the memory clinic was as affordable as possible, with minimal out-of-pocket expenses or to be free of charge (excerpt 3.5 h).

*“I think we’d be looking at patients with mild to moderate because we want to pick people up and we want to have them appropriately investigated and with access to treatment that’s there when they need it. So you know, we don’t want to wait until they’re moderate to severe, we want them to be still managing in the community at you know whatever we can optimise their level of functioning is.”* – 10GP (excerpt 3.5e)

## Discussion

This research captured local insights from GPs, seniors, community aged care workers, and community/local government representatives regarding the development of a new model of care for a multidisciplinary memory clinic. GPs had mixed attitudes towards their role in the assessment of cognitive impairment in their older patients resulting in a heterogeneous approach towards the assessment of MCI and dementia in primary care. Despite this, many GPs found it useful for patients to be diagnosed with MCI and dementia, indicating the need for assistance with diagnosis by secondary care. Whilst demonstrating an in-depth collective understanding of existing local dementia-specialised services and resources, participants discussed the need for improvement. This was largely due to wide-ranging access barriers including disparate, disconnected, and unclear pathways through services making it difficult for patients and carers to navigate; the need for a local, single point of contact, or 'one-stop-shop' for carers, patients, and health care providers was repeatedly articulated throughout. A range of expectations regarding the model of care for the new memory clinic were captured including clear and ongoing communication with GPs, the provision of diagnostic services, rapid and clear referral pathways, case management via a key worker, education, allied health and psychological support services, legal services, culturally sensitive and appropriate services, research participation opportunities, and an overarching focus on MCI and mild to moderate dementia. Participants described several barriers to memory clinic utilisation including transportation access, funding, awareness, and costs. Findings confirm the need for a new comprehensive multidisciplinary memory clinic in South Western Sydney that complements and integrates current dementia services and resources, and takes into account the local nuances of this diverse region [32].

The attitudes of GPs towards their role in the assessment of their patients for cognitive impairment varied greatly. Those who regularly assessed patients did so due to the high prevalence of cognitive impairment in the elderly or due to their own special interest in aged care, whilst others only assessed when there was clinical suspicion (as reported elsewhere [37, 38]). Consistent with previous studies, barriers to assessment included lack of time and a perception that patients would fear the results, damaging the therapeutic alliance [27, 28]. However, training GPs to identify early dementia does not necessarily translate to better management [39], indicating the need for any new dementia service to support GPs in their patient management role; this was also noted by GPs in the present study.

Most GPs saw the utility in a diagnosis so that families could better understand and manage the situation, plan for the future, and patients could begin treatment to help with symptoms. Yet the majority of GPs felt they needed assistance with the diagnosis from a secondary care specialist physician due to the challenges and consequences that making a diagnosis presents, time constraints, and the perception that only specialists can prescribe anti-dementia drugs. Others felt that a diagnosis was not useful as it caused patient distress and because dementia has no cure. Previous research has reported similar barriers [26], with one study reporting that nearly half of GPs across 12 Health Authorities in England and Wales did not find a diagnosis of dementia beneficial [40]. Missed and delayed diagnoses of dementia can result in significant harm, expense, and lost opportunity [41, 42]. In order to promote screening and early detection of dementia in primary care, efforts should be focused on removing barriers to diagnoses [37]. Providing additional support for GPs to boost confidence to manage patients post-diagnosis may facilitate this.

A sound understanding of local dementia resources and services for people with MCI and dementia were identified by community forum participants. Some satisfaction with current resources and services was identified by both GPs and community forum participants, however, the majority of participants articulated the need for improvement, and the addition of new dementia-specialised services with staff who had appropriate training, particularly with the provision of a dementia key worker. Prior research points to the benefits of a care coordinator (or key worker) to support the management of dementia for patients and families [43, 44]; clinical guidelines also recommend their provision [23].

Barriers to current services and resources include a lack of clarity, accessibility, fragmentation, long waiting times and costs, and navigability for the person with dementia, their carer, and the GP. Such service-issues have been reported elsewhere [45], and have been shown to contribute to caregiver stress [46]. Health Pathways has been recently implemented and its impact yet to be evaluated, but it is aimed at addressing some of the barriers to current services and resources described above. Interestingly, our findings (particularly those from community forum participants) differ to previous research which found that carers were not using dementia services due to a lack of awareness or perceived lack of need [47]. GPs also felt that there was limited communication between their practices and existing dementia services, resulting in a lack of understanding about potential support for patients, and no clear referral pathway. It is important that the model of care for the memory clinic takes these barriers into account, particularly in relation to the quality of information and dissemination methods.

Both GPs and community forum participants articulated that dementia resources available through the clinic or other means should focus on education for GPs, aged-care workers, seniors, and people with dementia/carers, improving dementia literacy (such as through public awareness campaigns focused on reducing dementia stigma), increasing awareness of available health pathways, and prevention of polypharmacy and medication misuse. The dementia services needed included case management via a key worker, home services, and carer support and respite. Care should be taken to widely promote the latter due to the previously reported lack of uptake with such services [47]. 

## Conclusions

Participants outlined a range of expectations on what services and features that the new memory clinic should offer, and potential barriers that should be avoided; these are summarised in Table 4. There was also an expectation from GPs that a new memory clinic should foster clear and continuous communication with them, from diagnosis through to ongoing management. They also felt that a list of the memory clinic’s services would have value to them and would promote referrals to a new clinic.

**Table 4:** Recommendations on services, resources, and features for the model of care for the proposed multidisciplinary memory clinic. Focus on patients with MCI, and mild to moderate dementia

(1) Services and resources: (a) Specialised diagnostic services promoting early and definitive diagnoses (b) Rapid access to care via brief referral processes and short waiting lists (c) Case management via a key worker (d) Access to allied health providers (e) Support groups for patients and carers (f) Links to community services (g) Medico-legal assistance (h) Advance care planning (i) Psychological support including counselling (j) Educational resources (k) Provide or refer to an outreach team (e.g., Community Geriatrics Service) (2) Features: (a) One-stop-shop (b) Multidisciplinary team (c) Easy referral process (particularly via fax or email) (d) Focus on improving integrated care through clear communication with GPs and stakeholders (e) Easily accessible (public transport, sufficient parking, free shuttle bus) (f) Culturally sensitive via multicultural and/or bilingual workers (g) Research participation opportunities including clinical trials (h) Affordable (i) Continuity of funding	

This study had several limitations. First, the sample engaged was largely community-based, and as a result, the views of hospital-based staff including specialist clinicians and health service managers were under-represented. Given that a memory clinic has the potential to positively impact hospital service delivery by reducing emergency department presentations, admissions, and inpatient length of stay (due to adequate diagnosis, management, and advance care planning) [10–13], it is essential that these views are taken into account. This will be addressed in a follow-up Delphi method study that engages an expert panel with equal representation from all stakeholder groups. Second, the findings from this study are difficult to disentangle from our research team’s preconceived notions of what the memory clinic’s model of care should look like. Unfortunately, a fundamental limitation of qualitative research is that the findings can often be influenced by the investigators’ personal biases [48]. Having said this, care was taken by the community forum facilitators (GZS, DK) and when interviewing GPs to present the interview schedule objectively and guided by the question prompts, reducing the influence of personal factors and improving consistency. Further, the specialist geriatrician (MIH) and GP (CE) on the research team was excluded from the forums to avoid confirmation bias. Findings should be interpreted in the context of the forum facilitators’ and interviewer’s expertise and gender (detailed in the methods) as recommended by the consolidated criteria for reporting qualitative research (COREQ) checklist [49]. Another limitation is that selection bias is possible with our GP cohort, that is, GPs interested in aged care/dementia may have been more likely to volunteer to be interviewed, and we may not have captured the views of less interested/engaged GPs. Further, differences in responses between community forum participants (e.g., community members vs. policy-makers) cannot be delineated as data were recorded and transcribed verbatim, and participants did not individually introduce themselves before speaking (as identities were already know to the facilitator). In addition, questions in relation to attitudes towards diagnosis and management of MCI and dementia were asked to GPs only, and not community forum participants. This was done intentionally for two reasons: (1) we expected GPs to have greater barriers towards diagnosis and management of MCI and dementia than community forum participants; and (2) due to the large number of community forum participants, the aim was to keep the discussion focused on service and resource provision, rather than community attitudes towards diagnosis. This should be explored further in future research.

In summary, this research gained the insights of the community, health workers, and policy makers to develop a model of care for a new memory clinic due to be located in South Western Sydney. The co-created model of care would address identified gaps in local dementia services and resources, focusing on supporting patients with MCI, and mild to moderate dementia with their diagnosis and management. Key aspects of the model highlighted repeatedly throughout include a 'one-stop-shop' that provides access to a dementia key worker for coordinated care, and education services for clinicians, people with dementia, and their carers. Findings not only confirm the need for a new multidisciplinary memory clinic, but importantly, operationalise the NHMRC’s Clinical Guidelines and Principles of Care for People with Dementia [23], and pave the way for a new 'gold standard' of memory clinic to be implemented locally, and upscaled nationally and internationally.

## Supplementary information

**Additional file 1.** Community forum qualitative interview schedule

## Data Availability

The datasets generated and analysed during the current study are not publicly available because data are qualitative in nature (recorded interviews and focus groups that were transcribed verbatim) and could potentially result in the reidentification of participants. Deidentified transcripts are available from the corresponding author on reasonable request.

## Abbreviations

ADLs: Activities of Daily Living;
CPD: Continuing Professional Development
GP: General Practitioner
MCI: Mild Cognitive Impairment
NHMRC: National Health and Medical Research Council
SD: Standard Deviation
SWSPHN: South Western Sydney Primary Health Network

## Footnotes

1 A new Primary Health Network initiative is Health Pathways, which is an online point of care clinical and referral information portal for primary care practitioners.
