# Supplementary material for: “We need a one-stop-shop”: co-creating the model of care for a multidisciplinary memory clinic with community members, GPs, aged care workers, service providers, and policy-makers
Source: BMC Geriatr. 2020 Feb 11;20:49. doi: 10.1186/s12877-019-1410-x (PMC7014614; doi:10.1186/s12877-019-1410-x)
Supplement: Supplementary file 1 — Additional file 1. Community forum qualitative interview schedule [file 12877_2019_1410_MOESM1_ESM.docx]

Community Forum Qualitative Interview Schedule

Expected audience: local health care providers and health care consumers.

Brief introduction to the project: There are currently over 353,800 people in Australia living with a diagnosis of dementia, and this number is expected to increase to nearly 900,000 by 2050. Macarthur will be the most affected region in NSW, with dementia prevalence rates in Campbelltown, Camden, and Macquarie Fields forecast to increase up to 460% by 2050. There is a clear need to develop novel, innovative, and cost-effective methods that will provide robust dementia-specialised clinical care in Macarthur to respond to the growing number of people who will be affected by dementia. Western Sydney University, together with partners in the South Western Sydney Primary Health Network, and South Western Sydney Local Health District are proposing to open a memory clinic to support the growing number of people with dementia and their families in the local region. The proposed memory clinic will offer a multidisciplinary model of care and will serve as a hub for dementia education, training, research, and clinical practice in South Western Sydney. The proposed services offered by the clinic include: specialised early diagnostic services, case management, education services (for patients, carers, and practitioners), linking patients to appropriate community services, access to allied health to support patients and carers through their journey with dementia, legal assistance and advanced care planning. We want to get an idea from you about what you think is needed for such a service.

1. What types of dementia-specific services are already available in the local area?

*Prompts:* Community-based services (e.g., day care, Alzheimer’s Australia dementia cafes and counselling services), DBMAS (Dementia Behaviour Management Advisory Service), specialist clinics (geriatricians/neurologists), aged care facilities/nursing homes.

2. How adequate are these current services in meeting the needs of the community?

3. What types of services for people with dementia and their families, and practitioners would you like to see more of in the local region?

4. What about resources for people with dementia and their families or practitioners? What types of resources do you think would be helpful?

5. Where or how would you like to access these services and resources?

6. What do people with dementia and their families in South Western Sydney need that they are not getting?

*Prompts:* Help with diagnosis, management, and/or advanced care provision and planning, support at home, social opportunities.

7. If there was a local memory clinic, what would you expect from such a service?

*Prompts:* Case management, rapid access to specialised early diagnostic services, education services (for patients, carers, and clinicians), linking patients to appropriate community services, access to allied health to support patients and carers through their journey with dementia, legal assistance and advanced care planning.

8. What are your potential issues with a memory clinic service like the one we are proposing?

*Prompt:* Refer to slide showing model of care and services provided.

9. Is there anything else you feel is important to highlight at this stage of the consultation process?

GP Qualitative Interview Schedule

Brief introduction to the project: South Western Sydney has some of the highest forecast increases in dementia prevalence rates across all of Australia. The aim of this project is to find out more about the current medical and community management of cognitive impairment and dementia in South Western Sydney, with the purpose of informing future service delivery and resource development for practitioners, patients, and government.

**Brief Questions (< 10 minutes):**

1. Do you regularly assess your older patients for cognitive impairment? Why? Why not?

2. Do you diagnose patients with MCI or dementia? Why? Why not?

3. Is it useful for patients to have a diagnosis of MCI or mild dementia? Why is this useful/not useful for the doctor or the patient?

4. Do you need any assistance with dementia/MCI diagnosis, management, and/or advanced care provision from other services?

5. What about patient or practitioner resources? Is there anything you can think of that would be helpful?

6. Are you familiar with any dementia-specialised services in the local area? What are these?

7. Where/how could local dementia services be improved?

*Prompts:* Diagnosis, management, advanced care provision, anything else?

8. Are there some patients you would like to refer to these services and do not? Why? Why not?

9. Is there anything that your patients with cognitive problems need that they are not getting?

**In-depth Questions (20 minutes):**

10. If your patients had access to a local memory clinic, what would expect from such a service?

🡪 Explore each service component GP raises with follow-up questions:

What value would this bring for you?

What are the advantages or disadvantages for you, the patient or their family?

Have you had experience with this type of service component before? What worked? What didn’t work?

11. I am not going to go through a few other possible services a local memory clinic could offer. Let’s start with ___________ (case management, rapid access to specialised early diagnostic services, education services [for patients, carers, and clinicians], linking patients to appropriate community services, counselling, access to allied health to support patients and carers through their journey with dementia, legal assistance and advanced care planning).

What value would ________ bring for you?

What are the advantages or disadvantages of _________for you, the patient or their family?

Have you had experience with this _________ before? What worked? What didn’t work?

12. Out of those services we’ve just been through, which are the 3 most important services that add the most value for you? Please rank them in order.

13. Would you refer patients to a local memory clinic? Why? Why not?

*Prompts* (if answers yes): What type of referral criteria would you expect (e.g., MCI, mild, moderate, severe dementia)? How would you like the referral process to work?

14. What are your issues, if any, with a memory clinic service?

15. Is there anything else you feel is important to highlight at this stage?
